# Ceribell EEG shortens seizure diagnosis and workforce time and is useful for COVID isolation

**DOI:** 10.1002/epi4.12474

**Published:** 2021-02-27

**Authors:** Marian P. LaMonte

**Affiliations:** ^1^ Ascension St. Agnes Hospital University of Maryland School of Medicine Baltimore MD USA

**Keywords:** COVID‐19, electroencephalography, status epilepticus

## Abstract

**Objective:**

To determine whether the portable Ceribell^®^ electroencephalograph (EEG) (Mountain View, CA) used for suspected status epilepticus (SE) can reduce time to diagnosis and on‐call workforce demands and whether it can be applied to patients in respiratory isolation.

**Methods:**

A multidisciplinary team developed a protocol for the use of the Ceribell EEG. The staff deploying the device, the attending physician, and the interpreting neurologist completed evaluation tools for each patient. Data maintained for quality and resource planning of 18‐channel electroencephalography ordered for suspected SE were used as controls. Times to diagnosis were compared by application of Welch‐Satterthwaite tests and workforce call‐in demands by Fisher's exact *t* test. We evaluated qualitative data related to the use of the EEG in COVID‐19 isolation rooms and on its technical aspects and acceptance by staff members.

**Results:**

The Ceribell EEG reduced diagnosis time (*P* = .0000006) and on‐call workforce demand (*P* = .02). The device can be used at any time of day in any hospital care area and has advantages in respiratory isolation rooms.

**Significance:**

Compared with a standard 18‐channel EEG, the Ceribell device allowed earlier diagnosis of SE and non‐SE conditions and reduced workforce demands. Due to the ease of its use and its simple components, which can be readily disinfected, it is advantageous for COVID‐19 patients in isolation.

**Key Points**

- The 8‐channel Ceribell EEG shortened the time to diagnosis of SE and non‐SE conditions compared with standard 18‐channel electroencephalography.
- It also reduced the frequency of requests for technologists to return to the hospital after hours, because the device was easily deployed by staff members who were already taking care of the patient.
- The assessment could be performed at any time of day and at any level of care (emergency department, ICU, floor nursing units), including respiratory isolation rooms for COVID‐19 patients, even those in the prone position.
- The rapid diagnosis of non‐SE conditions reduced risk by avoiding administration of unnecessary medications (some of which are in short supply) and the concomitant costs.

## 1 INTRODUCTION

Status epilepticus (SE) is an emergency neurologic disorder whose outcome is based on rapid diagnosis and treatment within a 30‐minute time frame, similar to the door‐to‐needle times for ischemic stroke.^1^ Shortening time to diagnosis is based on 1) clinical suspicion and 2) electroencephalographic interpretation consistent with the diagnosis. Five hundred nineteen adult patients for whom emergency medical services were called for the complaint of “seizures'' were transported to our 275‐bed teaching hospital with 83 000 emergency department (ED) visits per year between January 1, 2019, and January 1, 2020 (data from the Maryland Institute for Emergency Medical Services Systems, May 2020). Seizure emergencies in our hospital are assessed with a standard 10‒20 montage 18‐channel electroencephalograph (EEG) deployed by a small staff of technicians who provide 24/7 coverage. The device is space consuming and must be brought into isolation rooms. The after‐hour on‐call response time may be as long as 3 hours. Thus, meeting time‐to‐treatment windows in community hospitals, with their practical limits on resources, poses an important challenge for treating this neurologic disorder.

Another barrier to shortening time to treatment for SE is that literature on alternative diagnostic electroencephalograms that are FDA approved for SE remains scant. Therefore, few neurologist electroencephalographer interpreters have seen these alternative methods described in epilepsy‐specific publications.

Ceribell (Mountain View, CA) has developed an FDA‐approved EEG that can be used by staff members providing care to patients with a suspicion of seizure. This device is a reduced‐montage (8‐channel) circumferential EEG that produces tracings covering the frontal, temporal, and occipital areas. The electrodes are implanted in a soft, adjustable headband that is easily applied around the head and secured for electrode grip. Once impedances fall below 5000 ohms, the amplified signals are transmitted to a pocket‐size device that allows patient data to be entered, sonification of the frequency, and real‐time viewing of the tracing by the care providers. The device simultaneously uploads the study via WiFi to a HIPAA‐compliant cloud portal for remote viewing by the interpreter. Sonification provides the interpreter with audible data similar to the sound frequency during carotid ultrasonography.^2^

We compared the Ceribell EEG with the 18‐channel EEG in regard to time to SE or no SE diagnosis, to assess differences in technician on‐call needs, and to determine whether the device could be deployed in COVID‐19 isolation. And because the Ceribell EEG is new to our hospital system, we sought information on its technical performance and acceptability.

## 2 METHODS

The study was approved by our hospital administration and met criteria for waiver (for consent) from our institutional review committee.

The Ceribell company donated the devices for this study.

Preparations included collecting multidisciplinary input on study procedures followed by scheduled training sessions for the charge nurses and respiratory therapists who would be using the Ceribell device. These groups were chosen because of their integral involvement in patients’ care, especially those in respiratory isolation. We used an evaluation tool to collect data from the staff members using the device, the ordering attending physician, and the interpreting neurologist. We used a numerical system to track study information so as to maintain patient confidentiality.

We designed the evaluation forms to capture qualitative information in response to the following questions related to factors that are considered when adopting new technology (see Appendix 1):

1. Could the Ceribell EEG allow diagnosis of SE in all patient care areas, including the emergency department, the intensive care unit (ICU), the medical‐surgical‐neurological nursing floor, and respiratory isolation rooms?
2. Would the Ceribell EEG performed in a routine time of 20‐30 minutes be sufficient for stat diagnosis of SE/no SE or would adding time onto the study provide a different or additive diagnosis for the requesting provider and provide more information on typical artifacts, acceptability, and quality of acquired EEG data?
3. Would the Ceribell EEG provide diagnostic information for SE comparable to the information obtained from a standard 18‐channel EEG?
4. Would training and application be considered easy and acceptable by the nurses and physicians who are caring for the patient?
5. Can information about drug cost savings be gleaned from this comparison?

### 2.1 Algorithm for the use of the ceribell EEG

Our multidisciplinary team created the following algorithm for deployment of the Ceribell EEG in specific clinical scenarios:

An attending physician who suspects SE contacts the on‐call neurologist to provide clinical context so that the neurologist can determine whether the Ceribell EEG is the appropriate diagnostic modality. Then, the attending physician enters a STAT order for the Ceribell EEG. A nurse, respiratory therapist, or electroencephalographic technologist trained in the use of the Ceribell EEG applies the headband, enters data, ensures low impedances, and verifies that the tracing is being recorded. The on‐call neurologist receives a call back from this staff member to audit sonification of each hemisphere and to provide real‐time technical guidance to optimize the tracing being viewed through the portal. The neurologist informs the attending physician if the electroencephalographic information does or does not indicate SE.

The standard recording length (20‐30 minutes) is used for Ceribell EEG cases to conform to our routine for 18‐channel control cases. To assess technical conditions and recording quality in different patient areas and in isolation rooms, some Ceribell recordings were extended up to 2 hours. When clinically indicated and to compare diagnoses determined by Ceribell versus 18‐channel EEG, a standard 18‐channel EEG was obtained following the Ceribell EEG assessment in at least 50% of the patients. Each staff member who placed the Ceribell EEG, the attending physician, and the neurologist completed an evaluation form pertinent to their role in the process.

### 2.2 Definitions

1. Patient with clinically suspected SE: Any patient for whom a physician seeks EEG data to correlate with a clinical history and examination (including unresponsive state, minor movements, continued clinical seizing) when SE is considered.
2. Time to diagnosis of SE/no SE was recorded as the time between placement of the STAT request order and completion of the study (STAT studies are routinely interpreted immediately upon completion). In our facility, EEG studies typically run 20‐30 minutes (the exact time is determined by the interpreting neurologist). If the interpreting neurologist documented the time she called the attending physician with the diagnosis, we used that time for both cases and controls. We maintained the 20‐ to 30‐minute recording time since we desired a diagnosis of SE or no SE in a time window when intervention may improve outcome.
3. Requests for on‐call technician assistance were defined as STAT requests that were made after routine working hours, requiring the technician to return to the hospital.

### 2.3 Study group

Our study group consisted of 10 patients who were assessed with the Ceribell EEG.

### 2.4 Control groups

Forty controls were culled consecutively from our quality and resource planning database. This database captures all STAT 18‐channel EEG requests, for which we document time of study order, time to study completion, and technologist call‐in requirement. The 40 controls included patients with routine recording times and those who received long‐term continuous electroencephalography. Twenty controls were performed as stat routine 20‐ to 30‐minute EEGs, and twenty controls were performed as stat continuous long‐term EEGs. To eliminate bias due to differences in time to study completion between patients who had a routine 20‐ to 30‐minute EEG and those who had continuous studies having a time to completion well beyond 30 minutes, for analysis of time to diagnosis of SE/No SE, we used only the 20 stat routine time (20‐30 minute) controls. Because time to completion of study was not needed for the analysis of workforce (need to call in the technologist), the entire group of 40 controls was used.

### 2.5 Statistical analysis

For time to diagnosis, groups were compared with an unequal variance *t* test (Welch's *t* test and the Satterthwaite formula for degrees of freedom). The significance level was set at a *P* value of 0.05. For frequency of after‐hour EEG performance, groups were compared by Fisher's exact *t* test, also with the significance level set at a p value of 0.05. Mean and standard deviations were used for normally distributed data.

Qualitative information gleaned from the structured evaluation forms was categorized for descriptive analysis. Technical features included (a) technical and artifactual findings of Ceribell studies and their remedies, (b) product performance in various hospital patient care areas, including respiratory isolation, (c) quality of the recording compared with that of the full 18‐channel device, and (d) differences in interpretation between the Ceribell and the standard EEG. Acceptability features included (a) ease of learning to use the device, (b) acceptance by the treatment staff to learn a new patient care role, and (c) treatment team and interpreting neurologist satisfaction with a new procedure for providing STAT feedback for clinically suspected SE. Information on initiation or curbing of seizure treatments such as benzodiazepines, anticonvulsants, and intravenous anesthetics was reported.

## 3 RESULTS AND FINDINGS

Ten consecutive Ceribell EEG studies were performed between January 23, 2020, and March 13, 2020. Forty consecutive STAT 18‐channel EEGs obtained between July 22, 2019, and January 3, 2020, were used as controls. For the 10 Ceribell studies, the mean time to interpretation was 23.8 minutes (SD 9.66) (Table 1). For the twenty routine time 18‐channel studies, the mean time to diagnosis was 126.5 minutes (SD, 66.72) (*P* =.0000006 [95% CI: 133.91, −71.49]; Table 2).

**TABLE 1:** STAT Ceribell ‐ 10 Ceribell Patients (used for SE/no SE diagnosis, workforce time, and quality and technical measures)

Patient	Clinical information	Primary analysis measures		Quality and technical measures			
		TTD	Tech Called In	Unit	Performed by	Respiratory Isolation	SE by Ceribell
1	31 M SE vs pseudoseizure	:40	No	Neurology floor	EEG technician	N	N
2	56 M cerebral palsy, rhythmic twitching of left foot	:20	No	Emergency department	RN	N	Y
3	34 M cardiac arrest, tonic posturing of right upper extremity	:20	No	ICU	RN	N	N
4	66 M h/o stroke, acute altered mental status	:15	No	ICU	RN	N	N
5	57 M respiratory arrest, seizure‐like movements	:27	No	ICU	RN	Y	N
6	61 F respiratory arrest, prior craniotomy, acute altered mental status	:24	No	ICU	RN	Y	N
7	62 F protracted altered mental status	:18	No	ICU	RN	N	N
8	53 F Down syndrome, epilepsy, acute altered mental status	:41	No	ICU	RN	N	N
9	86 F cardiac arrest with prolonged twitching	:13	No	ICU	RN	N	N
10	66 F h/o epilepsy, prolonged altered mental status	:20	No	ICU	RN	N	N

TTD, time to SE/no SE diagnosis; Tech Called In, workforce time.

**TABLE 2:** STAT 18‐Channel EEG ‐ 40 Control Patients

Patient	Clinical Information	Primary analysis measures	
		TTD^a^	Tech Called In^b^
1	81 M acute altered mental status, lethargic	2:22	No
2	58 M h/o alcohol withdrawal seizure, prior SE	5:15	Yes
3	57 F h/o temporal lobe epilepsy, stroke, witnessed seizure	1:10	No
4	54 M possible overdose, found down, unresponsive	1:54	Yes
5	61 M more unresponsive, gaze deviation	1:54	No
6	39 M uncontrolled twitching	2:47	No
7	33 M acute altered mental status, eyelid fluttering, tremors	1:30	No
8	89 F h/o stroke, significant mental status change	1:02	No
9	65 M h/o seizure disorder, lethargic	1:28	No
10	38 M cardiac arrest	1:42	No
11	38 M cardiac arrest	3:01	Yes
12	78 M h/o seizure disorder, acute weakness, tremors	3:32	No
13	57 F prolonged episode of seizure‐like activity	1:38	No
14	62 F h/o metastatic cancer, acute weakness and dizziness	0:57	No
15	73 F h/o stroke, acute altered mental status	1:56	No
16	89 M found down, unresponsive	1:01	No
17	85 F acute dyspnea	2:06	No
18	75 F found sleepy and lethargic, respiratory failure	1:56	No
19	71 M acute altered mental status, lethargic	3:23	Yes
20	32 M h/o seizure disorder, SE	0:56	No
21	86 M cardiac arrest	*‐*	Yes
22	66 F h/o stroke, confusion, persistent weakness	*‐*	No
23	34 F cerebral palsy, frequent seizures	*‐*	Yes
24	78 F cardiac arrest	*‐*	No
25	76 M acute altered mental status	*‐*	No
26	66 M h/o traumatic brain injury, agitation, unresponsive	*‐*	Yes
27	61 M h/o multiple strokes, worsening weakness	*‐*	No
28	73 M h/o stroke, acute altered mental status	*‐*	Yes
29	54 M cardiac arrest	*‐*	No
30	81 F h/o stroke, facial twitching, slurred speech	*‐*	Yes
31	42 F acute altered mental status, respiratory distress, witnessed seizure	*‐*	Yes
32	74 M h/o seizure disorder, SE	*‐*	No
33	77 M acute altered mental status, witnessed seizure‐like activity	*‐*	No
34	75 F respiratory arrest, bilateral upper extremity shaking	*‐*	No
35	32 M h/o seizure disorder, traumatic brain injury, SE	*‐*	Yes
36	85 F cardiac arrest	*‐*	Yes
37	85 F cardiac arrest	*‐*	Yes
38	32 M h/o seizure disorder, SE	*‐*	Yes
39	23 F seizure attacks	*‐*	Yes
40	73 F acute altered mental status	*‐*	No

^a^ Control patients 1‒20 used for both time to SE/no SE diagnosis and workforce time (routine recording).

^b^ Control patients 21‒40 used for workforce time only (continuous recording).

Technologists were not called to the hospital after hours for any of the 10 Ceribell studies. They were called in to assist with 15 of the 40 control studies in which the 18‐channel EEG was used (*P* =.02 [95% CI: 0.000, 0.716]; Table 2).

In regard to qualitative questions, data from the evaluations revealed few technical issues. A few impedance issues were encountered and resolved by discussion between the neurologist and nurse until the impedance was below 5000 ohms. In some cases, resolution was achieved by simply repositioning the patient's head with a towel under the neck or adding more gel to the electrode caps. Artifacts known to be typical on 18‐channel EEG were seen. There was little to no sweat artifact. Movement artifacts and ECG artifacts were noted as an annotation by the nurse or EEG technician who was performing the study.

The device transmitted from any patient care area and in high‐intensity isolation rooms for COVID‐19 patients. The interpreting neurologist had no problems accessing the secure web portal using any device (desktop, I‐pad, cell phone) and could provide rapid diagnosis using both sonification and tracings. In two instances, the tracing upload to the secure portal was delayed 10 minutes. We surmised that these delays were caused by WiFi connectivity issues.

We found the simplicity of the device components advantageous. The headband was stored in the patient's room for reuse within 24 hours. During studies in isolation rooms, the recorder was placed in a small plastic bag, and, when the study was completed, it was easily cleaned using alcohol wipes. We encountered no barriers to the use of the Ceribell EEG, even when a patient was in a prone position for respiratory life support (the 18‐channel EEG is not practical for patients in this position). The staff evaluations indicated that training and deployment of this new technology and the related patient care tasks were acceptable. The average time to deploy and start recording was <10 minutes. Attending physicians’ impressions were universally favorable, especially for time to diagnosis and their ability to tailor treatment to the diagnosis.

Other important qualitative findings appreciated during this study to evaluate this new technology for future implementation in our facility included:

1. For those Ceribell EEGs that had a prolonged time of 2 hours to look for erosion of the Ceribell tracing by artifacts and impedance due to the prolonged time on the patient's head, there was no change in diagnosis by the interpreter whether sonification and tracing lasted 20 minutes or whether the study was prolonged for 2 hours. We acknowledge that our sample size is small.
2. To look for qualitative differences between the EEG types, for 7 of 10 patients, the Ceribell EEG was followed immediately by an 18‐channel EEG. In six of those seven cases, the interpretations of SE or no SE were the same; in the seventh case, the Ceribell EEG indicated SE and treatment with anticonvulsant was completed. The follow‐up 18‐channel EEG performed 30 minutes later did not detect seizures.
3. In two cases, the diagnosis based on the Ceribell EEG findings supported staff and family members in the decision to change the medical course from continued aggressive treatment to palliative care.
4. In patients with a clinical presentation suggestive of SE, only 1 of the 10 Ceribell recordings indicated SE. The rapid diagnosis of no SE in the other nine patients eliminated "blind" or “temporizing” treatment, which frequently consists of administration of benzodiazepines as a diagnostic trial when immediate electroencephalography is not possible. In addition to intravenous benzodiazepines, blind or temporizing treatment for SE may include anticonvulsants and intubation with intravenous anesthetic agents (eg, propofol, midazolam, ketamine) for presumed but not yet confirmed super‐refractory SE if electroencephalography will be delayed.

## 4 DISCUSSION

Indeterminate altered mental status and abnormal movements raise suspicion for SE with high frequency among ICU, emergency department, and hospitalized patients.^3^, ^4^, ^5^, ^6^ Decisions regarding the course of treatment rely on electroencephalographic data.

A number of practical challenges arise when trying to provide rapid data to the treating clinician, especially in a treatment window when intervention is known to improve outcomes from SE. Technologists and interpreters are not available around the clock in many community and rural hospitals. Space for bedside equipment is limited when patients require an array of supportive devices such as respiratory ventilators, intravenous machine pumps, vital signs monitoring equipment, and respiratory isolation carts. Additionally, these patients often need brain imaging studies, so provisions must be made to discontinue, then restart, 18‐channel electroencephalography to avoid the imaging artifacts that the metal EEG electrodes produce. The extensive protective gear necessary to limit staff members’ exposure to the coronavirus adds another layer of burden in treatment areas. These challenges must be surmounted to minimize delays in collecting electroencephalographic data and thereby balance the risks of undertreatment for SE against those associated with misdirected treatment when the diagnosis turns out not to be SE.

Researchers have attempted to reduce these burdens by assessing the applicability of reduced‐montage (eight‐channel) electroencephalography, constructing new paradigms with existing technologies (WiFi, Internet, security protocols), producing audible EEG signals, and linking these adaptations to patient‐specific, point‐of‐care EEG systems. Among them, we chose Ceribell to study because of its published sensitivity and specificity data.^7^, ^8^, ^9^

Both the primary analyses and the qualitative findings suggest a favorable profile of reduced circumferential montage EEG that can be deployed rapidly by the present patient care team and an EEG interpreter in attendance or remotely. Although this study had small numbers of patients, the differences between cases and controls were statistically impressive. The qualitative findings proved useful for acceptance of a new technology by hospital staff, reduced the need for unnecessary medications in 9 of 10 patients, and led to a change in course of care for 2 of 10 patients and their families. Shortening the time to SE or no SE diagnosis brings treatment time into a period known to improve patient outcomes from SE.

Limitations of this study include its nonrandomized comparisons. Having one interpreter review data collected from the Ceribell EEG and the subsequent standard 18‐channel EEG reduced inter‐interpreter bias. Important clinical limitations of the Ceribell device as compared with the 18‐channel EEG include the following:

1. The Ceribell EEG does not replace the standard 18‐channel EEG or long‐term continuous video electroencephalography because its reduced 8‐channel circumferential montage does not capture central and paracentral cranial areas. It does provide an additional tool in the care team's armament when SE is within the clinical differential.
2. This study utilized prospective cases but retrospective controls.
3. The Ceribell device does not have video capacity for the use by interpreters in making clinical correlations.

## 5 CONCLUSIONS

In this study, the Ceribell EEG shortened the time to diagnosis of SE and non‐SE conditions compared with standard 18‐channel electroencephalography and reduced the frequency of technologist call‐in requests. The Ceribell EEGs were easily deployed by staff members who were already taking care of the patient. The assessment could be performed at any time of day and at any level of care (emergency department, ICU, floor nursing units), including respiratory isolation rooms for COVID‐19 patients, even those in the prone position. This device is especially applicable to such patients, since the headband can be stored in the patient's room for reuse if clinical suspicion recurs, thus reducing cross‐contamination. The pocket‐sized data capture device can be placed in a sealed bag during use and then decontaminated when the assessment is complete. The rapid diagnosis of non‐SE conditions yields the positive outcomes of reducing risk by avoiding the administration of unnecessary medications (some of which are in short supply) and the concomitant costs. We recommend further studies on patient risk reduction and the financial aspects of care associated with the use of the Ceribell EEG.

## CONFLICTS OF INTEREST

I have no conflicts of interest to disclose.

## ETHICAL PUBLICATION STATEMENT

I affirm that the work described in this paper is consistent with the Journal's guidelines for ethical publication.

## APPENDIX 1 Evaluation Forms for Ceribell Technical and Quality Performance

A. Evaluation Form for Those Placing and Recording Ceribell studies (nurses, technologists, and respiratory therapists) (circle answers)

1. Reason for rapid response order:
  - Post cardiac arrest
  - TTM seizure surveillance
  - Altered mental status
  - CNS lesion
  - Status epilepticus suspected
  - Other: __________________
2. Patient location: ICU ED Floor bed Isolation
3. Person placing Ceribell: Nurse EEG tech Respiratory therapist
4. How long did it take to place Ceribell: <5 minutes 5‒10 minutes 10‒15 minutes >15
  - If >15 minutes, list complicating factor(s): ____________________________
5. How easy was Ceribell to place: Easy Medium Hard
6. Were templated or other annotations used during study? Yes No
7. Were medications and times given made into annotations? None All Some
8. Please provide technical and clinical feedback on your experience.

B. Evaluation Form for Those Determining Effectiveness of Ceribell for Clinical Treatment (requesting/ordering providers) (circle answers)

1. Reason for rapid response order:
  - Post cardiac arrest
  - TTM seizure surveillance
  - Altered mental status
  - CNS lesion
  - Status epilepticus suspected
  - Other: _____________________
2. Patient location: ICU ED Floor bed Isolation
3. Was the ordering process and deployment acceptable? Yes No
4. Did use of Ceribell shorten the time to diagnosis of SE or No SE? Yes No
5. Did use of Ceribell shorten your time to treatment? Yes No
6. Was the process useful for optimizing care for your patient? Yes No
7. Please provide technical and clinical feedback on your experience.

C. Evaluation Form for the Quality of Data and the Clinical Effectiveness of Ceribell (neurologist Interpreters) (circle answers)

1. Reason for rapid response order:
  - Post cardiac arrest
  - TTM seizure surveillance
  - Altered mental status
  - CNS lesion
  - Status epilepticus suspected
  - Other: _______________________
2. Patient location: ICU ED Floor bed Isolation
3. Overall quality of the data: Good Fair Poor
4. Did the length of the study produce a change in diagnosis? Yes No
5. Was the overall data recorded by Ceribell consistent with the data acquired by standard 18‐channel EEG? Yes No
6. Did annotations used provide useful information for interpretation? Yes No
7. Were medications and times given recorded in annotations? None All Some
8. Could a diagnosis of SE or no SE be made by the recording? Yes No
9. Did use of Ceribell shorten your time to diagnosis of SE or no SE? Yes No
10. Did use of Ceribell shorten the time to treatment? Yes No
11. Please provide technical and clinical feedback on your experience.
